# The pathogenic theory of homeostasis threshold deviation (HTD) in wide-range oscillatory physiological parameters: novel perspectives for hypertension and metabolic disease treatment

**DOI:** 10.1093/lifemeta/loaf029

**Published:** 2025-07-19

**Authors:** Wei Sun, Jin-Yu Sun, Xiangqing Kong

**Affiliations:** Department of Cardiology, The First Affiliated Hospital with Nanjing Medical University, Nanjing, Jiangsu 210029, China; Department of Cardiology, The First Affiliated Hospital with Nanjing Medical University, Nanjing, Jiangsu 210029, China; Department of Cardiology, The First Affiliated Hospital with Nanjing Medical University, Nanjing, Jiangsu 210029, China

**Keywords:** homeostasis threshold deviation, neural topological network, physiological homeostasis regulation, hypertension, metabolic diseases

## Abstract

Based on current research in neuroscience, systems biology, and clinical medicine, we propose a novel theoretical concept: the “Homeostasis Threshold Deviation (HTD) Theory of Wide-Range Oscillatory Physiological Parameters”. HTD posits that, when the external environment undergoes significant and sustained changes or when visceral signals exhibit long-term abnormalities, central nervous system (CNS) network topologies reset first, establishing a new range of physiological setpoints before peripheral organs adapt. This central reset leads to a deviation in the topological information network structure of central nuclei, resulting in the displacement of the original range of physiological parameters that become challenging to restore. This deviation further triggers passive adaption in peripheral organs, ultimately causing complex multi-organ diseases and severe organ dysfunction. By targeting the CNS threshold shift, HTD provides a novel path for precision medicine. That is, restoring original homeostatic setpoints could enable both early prevention and durable treatment of hypertension and metabolic disorders, achieving a “two birds with one stone” effect or even partially curing related conditions.

## Biological neural networks: scientific insights from theory-driven research

The homeostasis threshold deviation (HTD) theory is a hypothesis-driven biological concept developed from current neuroscience, systems biology, and clinical medicine knowledge. It shares similar conceptual structures with the early stages of neural network theory, which emerged from logical abstraction long before it could be experimentally tested. To contextualize this epistemological approach, we revisit the development of biological neural network theory, which demonstrates how abstract, logic-driven models can illuminate hidden mechanisms and guide future research.

**Figure 1:**
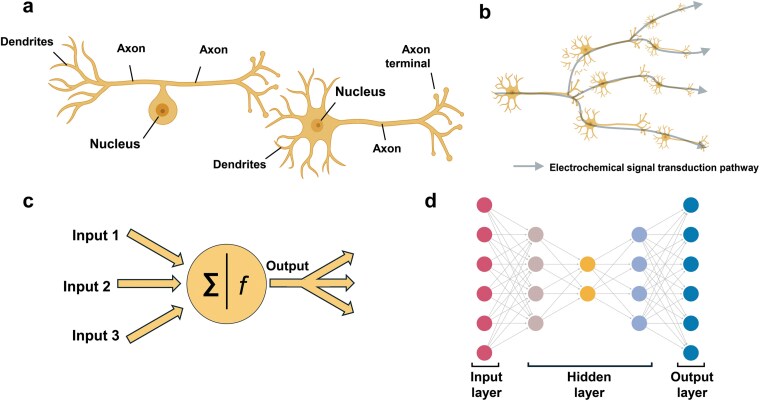
Schematic representation of biological and artificial neural networks. (a) Structure of a biological neuron. A neuron comprises dendrites, a cell body, an axon, and axon terminals. Dendrites receive external signals, which are integrated in the cell body and transmitted along the axon to other neurons. The left panel illustrates a pseudounipolar neuron, while the right panel shows a multipolar neuron. (b) Topological structure of a biological neural network. Neurons are connected via synapses, forming pathways for the transmission of electrochemical signals (indicated by arrows). (c) Structure of a single artificial neuron. An artificial neuron processes input signals by performing weighted summation (∑), applying a transformation via an activation function (*f*), and producing an output signal. (d) Structure of an ANN. An ANN comprises input, hidden, and output layers. These layers are interconnected through numerous artificial neurons, facilitating data input, feature extraction, and reconstruction.

Biological neural networks are complex systems consisting of numerous interconnected neurons. Although individual neurons possess simple structures and functions (Fig. 1a), their plastic synaptic connections enable the reliable reception and transmission of diverse electrochemical signals. These connections enable the collective functioning of neurons as a unified system, allowing them to perform complex tasks, such as perception, learning, memory, and decision-making [1] (Fig. 1b). The study of biological neural networks originated with abstract theoretical hypotheses derived from limited data, which were gradually validated through direct experimental evidence. Such a “theory-before-experiment” approach has established the foundation for our current understanding of neural networks.

In 1873, Bain proposed a groundbreaking theory that repeated activity strengthens neuronal connections, allowing learning and memory activaies [2]. This theory provided a concise but profound framework and guidance for following research on neural networks. However, due to the lack of advanced neuroscience techniques that time, Bain’s description of neural networks could not be directly validated, leading to widespread skepticism within the scientific community. Still, this theory introduced a foundational assumption that the brain’s learning and memory functions rely on neuron connectivity. It was not until the mid-20th century, with significant advancements in neuroanatomical techniques, that many neurons in the brain and their diverse synaptic connections became evident. Modern biological and physical technologies (e.g. microscopic imaging, neural tracing, and electrophysiological recording), combined with advanced computational and mathematical tools, have provided robust methods for validating neural network theories, progressively confirming Bain’s pioneering ideas [3–5].

The interplay between biological and artificial neural networks (ANNs) has further advanced this understanding. ANNs, which mimic the connectivity patterns and fundamental logic of biological neurons, are foundational to modern artificial intelligence revolution [6]. McCulloch and Pitts introduced a mathematical neuron model based on logical operations, capturing the “all-or-none” firing property of neurons [6]. Although initially simple, this model served as a foundational starting point for simulating the dynamic behavior of neural networks. Over the past decades, ANNs have evolved significantly through nonlinear mapping, feature extraction, and signal transmission into modern deep learning algorithms (Fig. 1c and d). These networks have been extensively applied across diverse computational fields and have also provided innovative tools and perspectives for exploring the dynamic properties of biological neural networks [7–9]. The integration of research on biological neural networks and ANNs has fostered a bidirectional process of “theory-experiment-revised theory”, driving innovation and deepening understanding in both fields.

## From set-point homeostasis to network-based regulation: reframing traditional theory

The body maintains a dynamic equilibrium within its internal environment (including the circulatory, metabolic, and immune systems) through self-regulation, which enables the adaptation to internal and external environmental changes and ensures the continuity of vital life activities [10]. This regulatory process, known as physiological homeostasis, is primarily controlled by the central nervous system (CNS). A classic example of homeostatic regulation is the “set-point theory of body temperature”, which identifies the hypothalamus as the central hub for temperature control. According to this theory, the hypothalamus maintains body temperature within a relatively stable range through feedback mechanisms, thereby ensuring proper metabolic function and overall physiological activity [11]. The traditional set-point theory posits that the CNS detects changes in both external and internal environments and transmits regulatory signals to peripheral organs, thus coordinating the functions of various systems. For example, in the circulatory system, blood pressure and heart rate rely on the dynamic balance between sympathetic and parasympathetic nervous system activities [12]. In the metabolic system, the hypothalamus monitors the body’s energy status, regulating food intake, fat storage, and energy expenditure to maintain glucose and lipid homeostasis [13, 14]. Similarly, the neuroimmune axis governs the innate immune system, which modulates immune cell activity to balance immune defense with anti-inflammatory responses [15].

Although traditional set-point theory established a foundation framework for understanding CNS-mediated homeostatic regulation, it primarily emphasizes the independent regulation of individual systems and over-relies on a linear, unidirectional control model from the CNS to peripheral organs. Therefore, this perspective neglects the integrative mechanisms across multiple systems and organs, and it also fails to capture the complex processes of information exchange and network remodeling within the CNS under diverse physiological and pathological conditions. Recent research demonstrates that the CNS does not depend solely on simple feedback regulation by isolated brain regions or neurons. Instead, it achieves dynamic integration across systems and organs through highly complex neuronal network connections spanning multiple brain regions [7, 16]. For instance, elevated body temperature increases metabolic rate and impacts inflammatory responses and immune activation, potentially altering circulatory system function. These findings suggest that homeostatic regulation requires coordinated interactions among multiple organ systems, facilitated by the CNS’s intricate network structure.

Recent advancements in ANNs have revolutionized artificial intelligence applications and offered novel perspectives for studying biological neural networks [9]. By simulating multilayered connections and topological structures among neurons, ANNs facilitate multidimensional information integration and dynamic regulation. These topological frameworks provide valuable insights into CNS networks’ hierarchical organization, information flow, and feedback mechanisms, thereby enriching our theoretical understanding of biological neural network topology. Elucidating how the CNS achieves multi-system homeostatic regulation through plastic topological information structures is fundamental for uncovering disease mechanisms and developing innovative therapeutic strategies in the following work.

## The pathogenic theory of HTD in wide-range oscillatory physiological parameters

**Figure 2:**
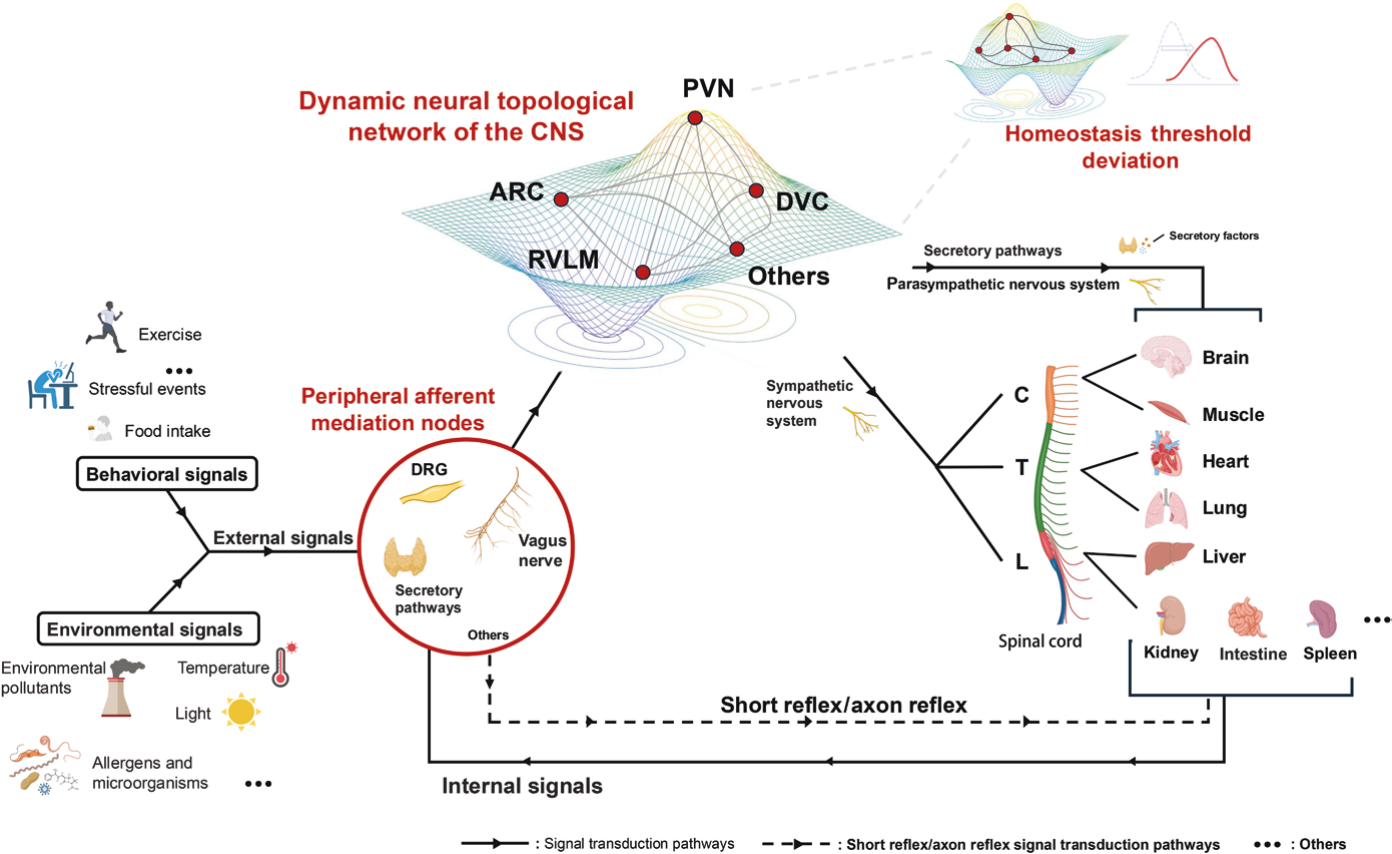
Topological structure of the homeostasis threshold in wide-range oscillatory physiological parameters. The CNS regulates homeostasis dynamically through a complex topological information network. External signals (e.g. behavioral and environmental inputs) and internal signals are transmitted to the dynamic neural topology network of the CNS via peripheral signal mediation nodes, such as the vagus nerve, dorsal root ganglia, or endocrine pathways. This dynamic neural network comprises multiple key nodes (e.g. PVN, DVC, ARC, and RVLM), which integrate incoming signals and form adaptive regulatory outputs through topological connections between neurons. The CNS topological network responds adaptively to different input signals by generating immediate changes, reflected in alterations in signal processing peaks and weights at the core nodes. These regulatory signals are then transmitted to peripheral organ systems, including the heart, lungs, liver, and kidneys, enabling dynamic regulation and overall coordination of circulatory, metabolic, and immune functions. This ensures that physiological parameters (such as blood pressure, glucose, and lipids) remain within the homeostasis threshold. When external or internal signals undergo sustained changes, a shift will occur in the central nuclei’s topological information network structure. This leads to an overall deviation of the physiological parameter range, making it challenging to return to the original baseline. The HTD further induces passive adaptive changes in peripheral organs, ultimately resulting in multi-organ complex diseases. PVN: paraventricular nucleus of the hypothalamus; DVC: dorsal vagal complex; ARC: arcuate nucleus; RVLM: rostral ventrolateral medulla.

Building on traditional set-point theory and our experimental findings [17], we propose the HTD Theory of Wide-Range Oscillatory Physiological Parameters (Fig. 2). Beyond traditional unidirectional feedback models, the HTD theory highlights that the CNS adapts before peripheral organs when the external environment undergoes significant and sustained changes or when long-term abnormalities arise in signals from visceral organs. Over time, the topological information network of central nuclei deviates, causing a deviation in the original range of physiological parameters that is difficult to restore to its baseline. This threshold shift induces passive adaptation in peripheral organs to the new set-point, leading to excessive strain, decompensation, and, ultimately, the development of multi-organ complex diseases and severe organ dysfunction.

The term “homeostasis threshold” encompasses two dimensions of variation: a specific threshold amplitude (A) and a range (R) representing the spatial extent across multiple brain regions and nuclei. This dual-dimensional framework captures the topological neural signal structures critical for physiological regulation. It should be noted that the regulatory function of the CNS is not limited to a single brain region or organ system; instead, it relies on highly complex topological information networks formed through neuronal connections across different brain regions or within the same brain region. The CNS integrates input from peripheral organs (such as the heart [18], liver [19], lungs [20], and gut [21]) via these distributed networks, enabling cross-organ regulation. The CNS then processes these inputs to generate adaptive regulatory signals that dynamically coordinate the functions of multiple systems, including circulation, metabolism, and immunity. For example, emerging research on the gut−brain axis can partly support this view, demonstrating that the gut microbiota communicates with the CNS via direct neural pathways (e.g. the vagus nerve) and indirect signaling mechanisms (e.g. immune and hormonal pathways) [22]. This bidirectional cross-talk between the gut and the brain modulates various physiological functions, such as mood regulation, immune responses, metabolism, and the local gut microbiota microenvironment, highlighting the involvement of the gut−brain axis in maintaining systemic homeostasis [21–23]. Besides, the cross-regulation between biological neural networks and the peripheral metabolic endocrine system should also be noted. For example, the hypothalamic−pituitary−adrenal axis and autonomic nervous system are classic key top-down regulatory pathways through which the CNS can modulate various physiological parameters [24]. Conversely, peripheral metabolic signals (including insulin, leptin, cytokines, etc.) can modulate neuronal activity and synaptic plasticity in the CNS [25, 26]. These bidirectional interactions form a tightly coupled network where peripheral metabolic endocrine signals can potentially alter central homeostasis thresholds and influence multi-organ regulation. Integrating this neuro−metabolic cross-talk further provides additional mechanistic foundation for the pathogenesis of complex chronic diseases.

Such coordination is essential in maintaining homeostasis, particularly for the wide-range oscillatory physiological parameters, such as blood pressure, lipids, and glucose. Through this integrative mechanism, the CNS adapts to internal and external environmental changes, ensuring that physiological parameters remain within the range of the homeostasis threshold. This dynamic regulation facilitates the long-term stability of the organism while permitting adaptive responses to fluctuating conditions. Ultimately, this cross-organ coordination underscores the theory’s central premise: the homeostasis threshold is not solely determined by individual systems but is a product of a complex, integrated regulatory network across multiple organ systems. The HTD theory can also be summarized in the following mathematical model:


$$\begin{array}{l} \Delta X_{patho}=max\left(0,\;\kappa \cdot \left(\left|\underset{i=1}{\overset{n}{\sum }}\;\omega_{i}\cdot f_{i}\left(S_{i},\;Y\right)\right|-A-\alpha \cdot R\right)\right) \end{array}$$

Here, ∆*X*_*patho*_ represents the pathogenic deviation in the physiological parameters from the normal range. *κ* is a constant that regulates the system’s sensitivity to the HTDs, reflecting the systemic responsiveness to input signals. *ω*_*i*_ is a set of weights assigned to each signal source (i.e. *S*_*i*_) in maintaining homeostasis and indicates the overall importance of *S*_*i*_ based on the different organs, brain regions, or systems. *f*_*i*_ (*S*_*i*_, *Y*) shows the regulation function of the *i*-th signal source (*S*_*i*_) on the homeostasis threshold, with *Y* representing the overall systemic state. Both internal and external factors are included. *A* represents the baseline fluctuation amplitude of a physiological parameter within its normal homeostatic range. *R* reflects the spatial extent of signal processing across brain nuclei and the connectivity scope of the central topological network. *α* is a constant to adjust the impact of the spatial range (*R*) on the deviation in the homeostasis threshold.

The HTD theory provides novel insights into the mechanisms underlying the development and progression of hypertension and metabolic diseases. In primary hypertension, increased vascular resistance and elevated blood pressure are not the root causes but consequences of altered homeostasis thresholds within circulatory homeostasis. Chronic stress or pathogenic factors (e.g. anxiety, obesity, or overeating) can disrupt peripheral signals, leading to a deviation in the CNS homeostasis threshold. This deviation precedes structural changes in the peripheral vasculature and is characterized by uneven sympathetic overactivation or dysregulation of endocrine feedback mechanisms. Once the homeostasis threshold has deviated, it becomes difficult to restore the original baseline, resulting in persistent hypertension and eventual structural abnormalities, such as increased vascular resistance or organ damage. A similar process underlies the development of metabolic diseases, where the hypothalamus, a key regulator of energy metabolism, integrates signals from the gastrointestinal tract, liver, pancreas, and adipose tissue to control appetite and energy expenditure [27]. Short-term overeating or prolonged high-fat diets can lead to a deviation in the hypothalamic homeostasis threshold, diminishing its sensitivity to leptin or insulin signals. This deviation not only directly increases appetite and reduces energy expenditure but also resists correction through simple behavioral interventions, disrupting metabolic balance and promoting the persistence of obesity and insulin resistance. Clinical symptoms of diabetes emerge when critical organs (such as the liver and pancreas) enter the decompensation stage. Thus, the challenging-to-reverse nature of HTD is a key mechanism driving the progression of chronic metabolic disorders to advanced, decompensated stages.

## Implications of the HTD theory for the treatment of hypertension and metabolic diseases

Hypertension and metabolic diseases are among the leading chronic non-communicable diseases worldwide [28, 29]. Traditional treatment strategies primarily target local functional regulation of peripheral organs. For instance, anti-hypertensive medications target blood vessels or kidneys to reduce blood pressure, anti-diabetic drugs enhance insulin secretion or improve peripheral insulin sensitivity to lower blood glucose, and lipid-lowering medications inhibit hepatic lipid synthesis to decrease blood lipid levels. However, these approaches overlook the systemic nature of disease onset and progression, failing to recognize the central role of the CNS in maintaining balance across multiple organ systems. As a result, discontinuation of these treatments often leads to the rapid recurrence of hypertension, hyperglycemia, and hyperlipidemia. The HTD theory provides a novel perspective on the pathological mechanisms underlying these diseases and introduces a new theoretical framework for precision medicine.

Beyond traditional treatment strategies, the HTD theory highlights the shifting from “local regulation” to “central integration” as a key direction for future therapies. Managing hypertension and metabolic diseases should not be restricted to targeting single organs or signaling pathways. Still, it should prioritize how the CNS reshapes overall metabolic homeostasis and intervenes to delay or block pathological deviations in the homeostasis threshold. Our recent research has demonstrated that perirenal fat contributes to the development of various types of hypertension by modulating the activity of L1-L2 dorsal root ganglion neurons [17]. Inhibiting afferent neural signals from perirenal fat could significantly improve deviations in the central homeostasis threshold, allowing a single intervention to achieve long-term blood pressure reduction without disrupting physiological blood pressure regulation. These findings suggest that targeting or blocking key peripheral signaling pathways to restore central HTD could be an effective therapeutic strategy. This approach holds the promising potential to simultaneously manage hypertension and metabolic diseases, achieving a “two birds with one stone” effect or potentially curing related conditions. To validate this concept, our research group is conducting two multi-center, blinded, randomized, sham-controlled clinical trials (NCT06018493 and NCT06283758). These trials aim to evaluate the therapeutic potential of focused ultrasound targeting fat in the lower renal pole to block or reverse deviations in the blood pressure homeostasis threshold. The studies will assess the clinical feasibility of this approach for treating primary hypertension and lipid metabolism disorders.

In conclusion, the HTD theory provides a novel theoretical framework for understanding the mechanisms underlying complex diseases and developing therapeutic strategies. Restoring the normal range of the homeostasis threshold and reestablishing systemic homeostasis could enable earlier, more comprehensive, precise, and sustained interventions for disease management.

## Limitations of the HTD theory

Although the HTD theory offers valuable theoretical guidance and inspiration for future research in related fields, limitations should be mentioned. Much like the challenges Bain faced when proposing the neural network theory in 1873, the HTD theory is limited by technological constraints and remains fully validated by experimental data. While modern techniques allow for recording electrophysiological signals from individual neurons or specific brain regions, capturing the instantaneous electrochemical signals and dynamic topological changes of billions of neurons across the whole CNS remains an unresolved technical hurdle. As a result, the HTD theory cannot yet be directly validated experimentally and relies on future advancements, particularly in high-resolution molecular imaging of brain neurons and real-time, whole-brain electrophysiological signal recording. Moreover, the HTD theory posits a dynamic and interconnected network between the CNS and peripheral organs, but the full complexity of these interactions may be underestimated. In establishing the current model, qualifying the resident factors that complicate the implementation of universal therapeutic strategies, such as genetic variability, environmental influences, and individual physiological differences, is challenging. Besides, as a broad conceptual model of disease development, the HTD theory may oversimplify the interactions between different systems and their response to physiological or pathological changes. The theory should benefit from further refinement to account for specific pathophysiological mechanisms in various diseases apart from hypertension and metabolic diseases.

## Perspectives

The HTD theory of wide-range oscillatory physiological parameters is a theoretical biological framework developed through logical synthesis, deduction, and abstraction based on existing evidence from neuroscience, systems biology, and clinical medicine. The HTD theory highlights that the CNS continuously receives input signals from peripheral systems and dynamically integrates information from multiple peripheral organs through complex topological information networks to maintain overall functional homeostasis. This theory surpasses the limitations of traditional “single-system regulation” models, providing a novel perspective on the mechanisms underlying hypertension and metabolic diseases. It provides a promising theoretical foundation and technical targets for early diagnosis, targeted interventions, and precision treatments.

Like Bain’s neural network theory, which was gradually validated decades after its proposal, we believe that technological advancements will enable future researchers to advance along the dual pathways of theoretical exploration and experimental validation. Currently, the integration of artificial intelligence (AI) with the HTD theory has provided a promising opportunity to deepen our understanding and application of this framework in personalized disease prediction. The advanced computational capabilities allow AI to analyze complex physiological data and capture the dynamic nature of homeostasis thresholds across multiple organs and brain regions. By examining individual health profiles, AI can identify deviations in homeostasis thresholds, providing a more tangible representation of the HTD theory, thus making it more applicable in clinical settings. AI can uncover subtle patterns and early indicators of HTDs through machine learning and data-driven models, enabling early disease prediction before clinical symptoms emerge. Accordingly, AI can aid in advancing our understanding of the HTD theory and holds significant promise for enabling precise, individualized disease prediction and intervention strategies. Moreover, digital human models can combine multi-organ physiological data (e.g. cardiovascular, metabolic, neural, and endocrine information) to simulate the dynamic changes of the human body. By being updated with real-time physiological parameters, digital human models provide an additional tool to reveal the complex interactions between systems and explore effective strategies to intervene before the disease progresses [30]. Ultimately, such progress may help elucidate the dynamic regulatory mechanisms of central neural networks, bridging the gap between theoretical insights and experimental validation.
